# Definition of Herpes Simplex Virus Type 1 Helper Activities for Adeno-Associated Virus Early Replication Events

**DOI:** 10.1371/journal.ppat.1000340

**Published:** 2009-03-13

**Authors:** Nathalie Alazard-Dany, Armel Nicolas, Aurélie Ploquin, Regina Strasser, Anna Greco, Alberto L. Epstein, Cornel Fraefel, Anna Salvetti

**Affiliations:** 1 INSERM U758, Lyon, France; 2 Ecole Normale Supérieure de Lyon, Lyon, France; 3 IFR128 BioSciences Lyon-Gerland, Lyon, France; 4 Université de Lyon, UCB-Lyon 1, Lyon, France; 5 Institute of Virology, University of Zurich, Zurich, Switzerland; 6 Université de Lyon, Lyon, France; Université Lyon 1, Lyon, France; CNRS UMR5534, Centre de Génétique Moléculaire et Cellulaire, Villeurbanne, France; Marie Curie Research Institute, United Kingdom

## Abstract

The human parvovirus Adeno-Associated Virus (AAV) type 2 can only replicate in cells co-infected with a helper virus, such as Adenovirus or Herpes Simplex Virus type 1 (HSV-1); whereas, in the absence of a helper virus, it establishes a latent infection. Previous studies demonstrated that the ternary HSV-1 helicase/primase (HP) complex (UL5/8/52) and the single-stranded DNA-Binding Protein (ICP8) were sufficient to induce AAV-2 replication in transfected cells. We independently showed that, in the context of a latent AAV-2 infection, the HSV-1 ICP0 protein was able to activate *rep* gene expression. The present study was conducted to integrate these observations and to further explore the requirement of other HSV-1 proteins during early AAV replication steps, i.e. *rep* gene expression and AAV DNA replication. Using a cellular model that mimics AAV latency and composite constructs coding for various sets of HSV-1 genes, we first confirmed the role of ICP0 for *rep* gene expression and demonstrated a synergistic effect of ICP4 and, to a lesser extent, ICP22. Conversely, ICP27 displayed an inhibitory effect. Second, our analyses showed that the effect of ICP0, ICP4, and ICP22 on *rep* gene expression was essential for the onset of AAV DNA replication in conjunction with the HP complex and ICP8. Third, and most importantly, we demonstrated that the HSV-1 DNA polymerase complex (UL30/UL42) was critical to enhance AAV DNA replication to a significant level in transfected cells and that its catalytic activity was involved in this process. Altogether, this work represents the first comprehensive study recapitulating the series of early events taking place during HSV-1–induced AAV replication.

## Author Summary

The Adeno-Associated Virus (AAV) is a human parvovirus that is widely used as a recombinant vector for gene transfer in animal studies and clinical trials designed to treat acquired or inherited genetic diseases. Wild type AAV is defined as a defective virus because it requires the presence of a helper virus to efficiently replicate. Although many viruses can provide helper functions to AAV, only those of Adenovirus were extensively studied, leading to the generation of molecular tools for recombinant AAV vector production. This study focuses on the helper activities of another virus, the Herpes Simplex Virus type-1 (HSV-1). We demonstrate that nine HSV-1 proteins are able to fully and efficiently support the early steps of AAV replication. As such, this study provides new information critical for the understanding of AAV replication and also opens the way for new biotechnological developments in the field of recombinant AAV vectors.

## Introduction

Adeno-Associated Virus (AAV) type 2 is a human parvovirus that is widely used as a vector for gene transfer. Its 4.7 kb single-stranded DNA genome contains two open reading frames, *rep* and *cap*, flanked by Inverted Terminal Repeats (ITRs) that serve as origins of DNA replication. Four Rep proteins are produced from the *rep* gene by two different promoters and splicing patterns. The major Rep78 and Rep68 proteins display DNA-binding, endonuclease, and helicase activities that are essential for AAV genome replication. Three structural proteins (VP1/2/3) are produced by the *cap* gene and assemble to form the capsid [1]. Productive replication of wild-type (wt) AAV depends upon the presence of a helper virus whereas, in its absence, AAV enters latency. During this phase, the viral genome is maintained in an episomal or integrated form and is transcriptionally silent. Upon re-activation by infection with a helper virus, the set of events leading to the production of infectious particles consists of the early activation of the *rep* gene, the rescue of the AAV genome and finally the generation of replicative DNA forms that also serve as templates for Rep and Cap protein synthesis [2].

Early studies identified several different viruses as able to display helper activities for AAV growth including Adenovirus (Ad) and Herpes Simplex Virus type 1 (HSV-1). The helper functions of Ad were extensively characterized and demonstrated to be supplied by the E1a, E1b-55K, E2a, and E4Orf6 genes, and by the VA RNAs [3]. Their main effects involve the induction of AAV gene expression and viral genome replication. Importantly, the DNA polymerase activity responsible for AAV replication in the presence of Ad was demonstrated to be of cellular origin [4].

In contrast, the helper functions provided by HSV-1 have been less extensively characterized. Early studies performed using sub-genomic fragments of the HSV-1 genome identified several immediate-early and early HSV-1 genes involved in the helper effect [5]. However, because immediate-early factors are essential for the expression of all subsets of HSV-1 genes when native promoters are used, distinguishing between direct or indirect helper effects was difficult. A more detailed analysis was conducted by Weindler *et al.* who demonstrated replication of transfected AAV-2 genomes using four HSV-1 genes coding for the helicase/primase (HP) complex (UL5/UL8/UL52) and the single-stranded DNA Binding Protein (ICP8) (encoded by the UL29 gene) [6]. Further work showed that, upon cotransfection of UL5/UL8/UL52, and UL29 expressing constructs, with a plasmid containing the wild-type AAV genome, AAV DNA replicated in discrete nuclear foci that co-localized with ICP8 and cellular replication protein A (RPA) [7],[8]. In this context, the UL52 primase activity was demonstrated to be dispensable whereas, the UL5 helicase activity was required [9]. Based on these results and on previous studies on the formation of HSV-1 replication centers, the HP proteins were proposed to constitute a scaffold that further recruits ICP8, Rep proteins, and AAV DNA. This model assumed that AAV DNA replication, which starts from a self-primed DNA template would then be conducted by the combined effects of Rep proteins, the HSV-1 helicase, ICP8, and cellular factors including the DNA polymerases.

Two questions were not addressed in those studies. The first concerned the role of HSV-1 DNA polymerase. Indeed, even if it was clear that the HP complex and ICP8 could induce AAV replication, the level achieved was less than 1% of that observed in cells infected with wt HSV-1, suggesting that some critical factors involved in DNA replication were missing [9]. A potential role for the HSV-1 DNA polymerase had been previously suggested by studies indicating that addition of phosphonoacetic acid (PAA) greatly reduced AAV replication during co-infection with HSV-1 [10]. In addition, the involvement of the HSV-1 DNA polymerase was further suggested by Ward *et al.* who demonstrated that this viral complex could replicate AAV DNA using *in vitro* replication assays in the absence of cellular factors [11]. However, despite these observations the role of the polymerase was not re-evaluated *in vivo* by adding it to the minimal set of HSV-1 helper factors.

The second unresolved question concerned the identification of other HSV-1 helper proteins potentially involved in regulation of AAV *rep* gene expression, a step essential to drive the virus into the replication cycle, as exemplified by the helper effect provided by the Ad E1A protein [12]. During natural AAV infection, the viral genome is either introduced into the nucleus as a single-stranded linear molecule by infection, or it is already present in a latent, integrated form. In both situations a viral transactivator is required to induce Rep protein synthesis. An initial study toward identifying HSV-1 transactivating factors was performed by Geoffroy *et al.* who demonstrated that the immediate-early ICP0 protein, which is known for its ability to activate viral gene expression, could also activate the transcription of the *rep* gene in cells latently infected with wt AAV2 [13]. However, those studies also revealed that other factors were still missing since when cells were infected with an HSV-1 mutant with a deletion of the ICP0 gene a low level of Rep proteins could still be detected (Figure S3). In addition, ICP0 expression alone was neither sufficient to induce Rep synthesis at levels similar to those found during wt HSV-1 infection, nor able to activate the expression of the AAV *cap* gene.

The present study was conducted to integrate the above observations and to further explore the requirement for other HSV-1 proteins during early AAV replication steps, i.e. *rep* gene expression and AAV DNA replication. Using a cellular model that mimics AAV latency and composite constructs encoding several immediate-early and early HSV-1 proteins expressed under the control of heterologous promoters, we first confirmed the role of ICP0 for *rep* gene expression and further demonstrated a synergistic effect of ICP4 and, to a lesser extent, of ICP22. Conversely, the other HSV-1 immediate-early protein tested, ICP27, was shown to have an inhibitory effect. Second, our analyses indicated that the effect of ICP0, ICP4 and ICP22 on *rep* gene expression was essential for the induction of AAV DNA replication in conjunction with the HSV-1 HP complex and ICP8. Third, and most importantly, our results indicated that the HSV-1 DNA polymerase complex (UL30/UL42) was critical to enhance AAV DNA replication to a significant level in transfected cells and that its catalytic activity was involved in this process. Altogether this work represents the first comprehensive study recapitulating the series of early events that take place during HSV-1-induced AAV replication.

## Results

### The ICP0, ICP4, and ICP22 immediate-early HSV-1 proteins cooperate to activate AAV *rep* gene expression from a latent integrated form

**Figure 1:**
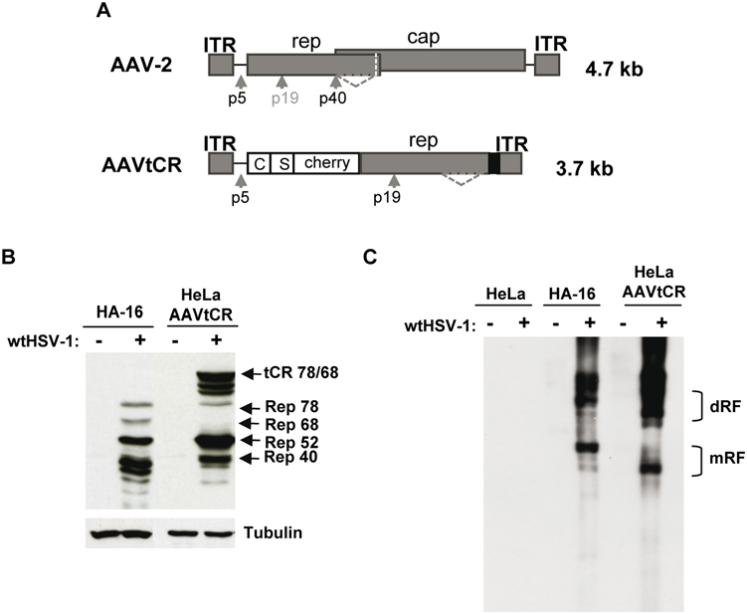
Characterization of the HeLaAAVtCR cell clone. (A) Schematic representation of the wt AAV-2 genome and of the AAVtCR derivative. In the AAVtCR construct the *rep* gene is fused at its N-terminus with a sequence coding for the calmodulin and streptavidin binding peptides (C and S) and the fluorescent mCherry protein. p5, p19, and p40 are the AAV promoters and the dotted line indicates the alternative splicing in the *rep* gene. The black box indicates the BGH polyA. (B) Synthesis of Rep proteins in HSV-1-infected HeLaAAVtCR and HA-16 cells. The cells were infected or not with wt HSV-1 at a multiplicity of infection (MOI) of 5 plaque forming units (pfu)/cell and analyzed for the synthesis of Rep proteins by Western blot. tCR indicates the tagCherryRep78 and 68 proteins. (C) Analysis of AVVtCR genome rescue and replication. Replication was assessed by Southern blot using a rep probe. mRF and dRF indicate the monomeric and dimeric replicative forms, respectively.

For this study we used a HeLa cell clone (HeLaAAVtCR) that contained integrated in its DNA a modified AAV genome composed of the AAV-2 ITRs flanking a *rep* gene in which the *rep* ORF was fused at its 5′ terminus with the fluorescent mCherry coding sequence and two affinity tags (Figure 1A). The analysis of this cell clone indicated that it contained approximately three AAVtCR genome copies per cell with at least one of these copies integrated into the AAVS1 locus (Figure S1). As previously observed with latently infected HA-16 cells, infection of these cells with wt HSV-1 led to the synthesis of the four Rep proteins, two of which had the expected higher molecular weight due to their fusion with mCherry and the two tags (Figure 1B) [13]. Most importantly, HSV-1 infection also led to the formation of AAV replicative forms, thereby indicating that the fused Rep proteins (tCR78 and tCR68) were fully functional (Figure 1C). Further studies also showed that the fused Rep proteins were fluorescent and localized to the nucleus. These analyses demonstrated that the HeLaAAVtCR cells constituted an appropriate model of AAV latency that could be used to study the early steps of AAV activation without any interference from the presence of the *cap* gene and the synthesis of AAV particles [14].

**Figure 2:**
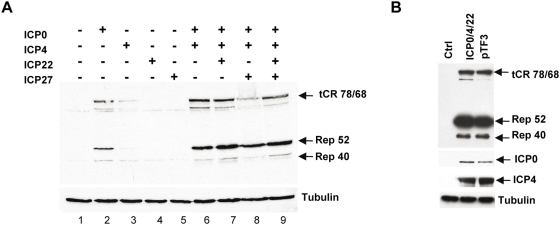
Analysis of Rep protein synthesis upon transfection with plasmids encoding HSV-1 immediate-early proteins. HeLaAAVtCR cells were transfected with the indicated plasmids and harvested 48 h later. (A) Cell lysates were analyzed by Western blot with an anti-Rep antibody (303.9) and, after stripping of the membrane, with an anti-tubulin antibody. (B) Comparison of Rep synthesis following either co-transfection with ICP0, ICP4, and ICP22 expressing plasmids or with the pTF3 construct. After analysis with an anti-Rep antibody, the membranes were stripped and re-probed with anti-ICP0, anti-ICP4, and anti-tubulin antibodies.

**Figure 3:**
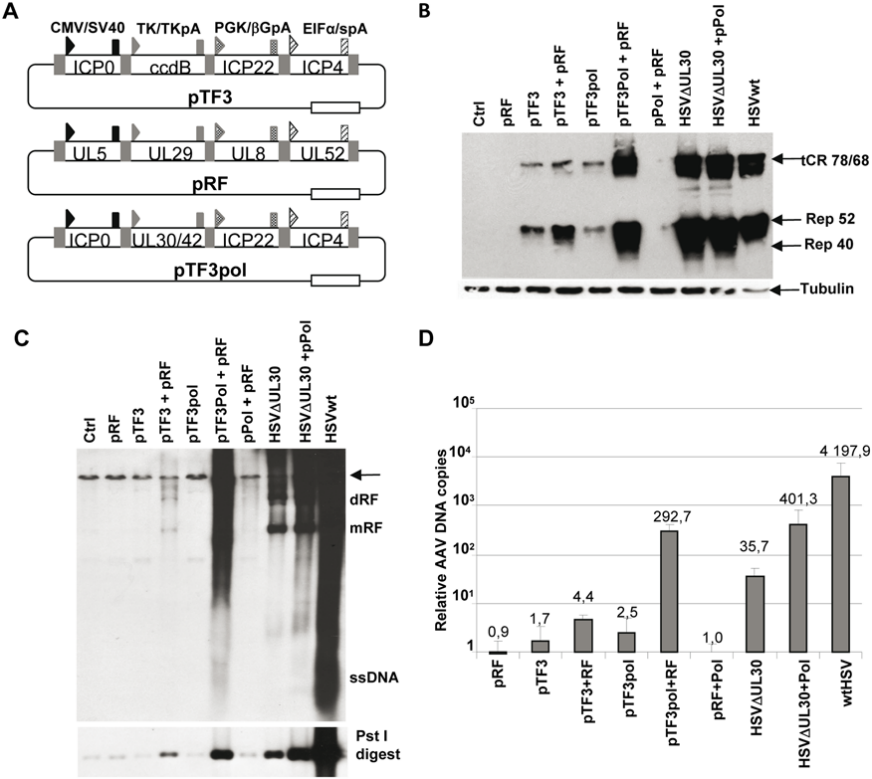
Induction of Rep protein synthesis and AAVtCR genome replication by combinations of HSV-1 immediate-early and early genes. (A) Schematic view of the HSV-1 constructs pTF3, pRF, and pTF3Pol. Each plasmid encodes three to four HSV-1 orfs expressed under the control of heterologous promoters and polyA signals. The arrow heads indicate the following heterologous promoters: CMV (black), TK (light grey), PGK (dark grey), short eIF1-α (striped box). The rectangles correspond to the following polyA signals: SV40 (black), TK (light grey), β-Globin (dark grey), and synthetic polyA (striped box). The grey areas between each gene correspond to the Gateway® recombination signals used to obtain these constructs. CcdB is a selection marker used to screen bacterial colonies. The white box indicates the ampicillin resistance gene. (B) HeLaAAVtCR cells were either transfected with different combinations of HSV-1 derived plasmids, or infected with wt HSV-1 or HSVΔUL30 (MOI of 5 pfu/cell). (C) Analysis of AAVtCR genome replication. Genomic DNA was extracted and analyzed by Southern blot with a rep probe. Upper image: undigested DNA; lower image: Pst I digested DNA. Digestion with Pst I was used to resolve all the AAVtCR replication products into a unique 1.4 kb band. (D) Analysis of AAVtCR genome replication by qPCR using primers located in the AAV *rep* gene. The quantity of rep sequences in each condition was determined as described in the Materials and Methods and presented as the fold enrichment relative to a cellular genomic sequence on chromosome X. The data shown are mean values with error bars representing the standard error of the mean for three independent experiments.

The HeLaAAVtCR clone was first used to investigate the effect of HSV-1 immediate-early factors on *rep* gene activation. Our previous study had indicated that the HSV-1 ICP0 protein was able to induce AAV *rep* gene expression from a latent integrated wt AAV genome [13]. Accordingly, transfection of the HeLaAAVtCR cells with a plasmid expressing ICP0 under the control of a CMV promoter led to the synthesis of Rep proteins (Figure 2A). Transfection of HeLaAAVtCR cells with plasmids expressing either ICP4 or ICP27 or ICP22, revealed that only ICP4 was able to activate *rep* gene expression alone, although to a lesser extent than ICP0. More interestingly, a clear synergistic effect of ICP4 and ICP0 on *rep* gene activation was also observed. Conversely, co-expression of ICP27 with ICP0 and ICP4 decreased the level of Rep proteins, in particular tCRep78/68 and Rep 40, whereas co-expression of ICP22 increased the level of Rep 52 and 40, in a very low but reproducible manner (Figure 2A) (Figure S6). Similar results were observed in cells transiently infected with wt AAV-2 particles (Figure S2). We concluded from these experiments that ICP0, ICP4 and ICP22 were sufficient to induce an optimal level of Rep proteins. Therefore, a plasmid combining the three expression cassettes coding for ICP0, ICP4 and ICP22 was constructed using a Gateway reaction (pTF3) (Figure 3A). Transfection of this construct induced AAV Rep protein synthesis at a level comparable to that observed upon co-transfection of the three individual plasmids encoding for ICP0, ICP4, and ICP22 (Figure 2B). Altogether these results confirmed the previously documented trans-activating activity of ICP0 on *rep* gene expression and further demonstrated a synergistic effect of ICP4 and, to a lesser extent, of ICP22, as well as an inhibitory effect of ICP27.

### *Rep* gene activation by ICP0, ICP4, and ICP22 is essential to induce AAV DNA replication in the presence of UL5/UL8/UL52 (HP) and ICP8

Previous studies indicated that the subset of HSV-1 genes able to provide helper functions for AAV DNA replication included ICP8 and the HP complex [6],[8],[9]. Therefore, after having shown that ICP0, ICP4, and ICP22 expressed by the pTF3 plasmid cooperate to activate *rep* gene expression, we determined their effect on AAV DNA replication either alone or in association with the previously identified HSV-1 helper replication factors. For this, the UL5/8/52 and UL29 genes were cloned as independent expression cassettes in a single plasmid, pRF, using the Gateway technology (Figure 3A). Transfection of the pRF plasmid into HeLaAAVtCR cells indicated that expression of these HSV-1 replication factors alone was not sufficient to induce Rep synthesis or AAV DNA replication (Figure 3B and 3C). In contrast, transfection of the pTF3 plasmid induced Rep protein synthesis but no AAV replication. More importantly, co-transfection of pTF3 with pRF increased Rep synthesis and generated detectable replicative forms. These results indicated that the three HSV-1 transactivating factors were essential to induce AAV replication in association with the HP and ICP8 proteins. Nevertheless, in these conditions the level of Rep proteins and AAV DNA replication remained very low compared to the level observed in cells infected with HSV particles.

### Effect of the HSV-1 polymerase

**Figure 4:**
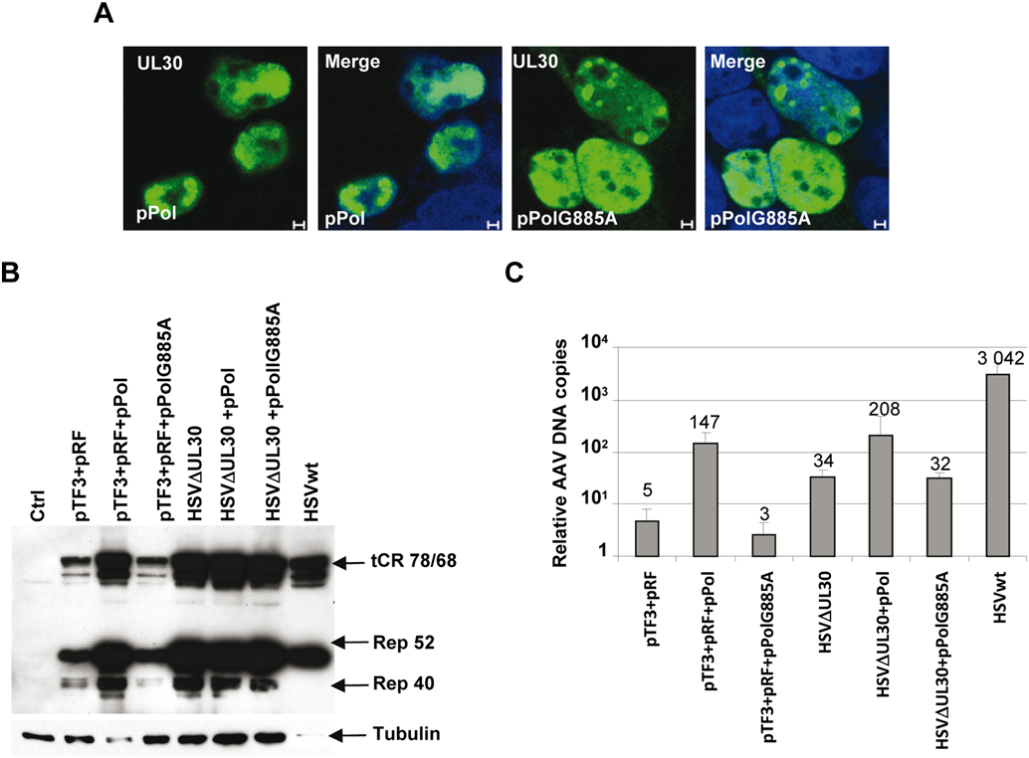
Effect of a mutated HSV-1 DNA polymerase on Rep protein synthesis and AAV DNA replication. (A) Intracellular localization of the wt and mutated HSV-1 polymerase in HeLa cells. HeLa cells were co-transfected with pTF3 and pRF and either the pPol or pPolG885A plasmid. 48 h post-transfection the cells were fixed, stained with an anti-UL30 antibody and observed under a confocal microscope. Cell nuclei were stained with Topro3. Bars: 2 µm (B) Analysis of Rep protein synthesis. HeLaAAVtCR cells were transfected with the indicated plasmids and, where indicated, infected 24 h later with the appropriate HSV-1 strains at an MOI of 2 pfu/cells and harvested 24 h later. Cell lysates were analyzed by Western blot with an anti-Rep antibody (303.9) and, after stripping of the membrane, with an anti-tubulin antibody. (C) Genomic DNA was analyzed by qPCR using primers located in the AAV *rep* gene to measure AAVtCR replication. The amount of rep sequence in each condition was determined as described in the Materials and Methods and presented as the fold enrichment relative to a cellular genomic sequence on chromosome X. The data shown are mean values with error bars representing the standard error of the mean for three independent experiments.

Because previous studies had documented a controversial role of the DNA HSV-1 polymerase in AAV replication, we then tested its impact on the induction of AAVtCR genome replication [6],[10],[11]. For this, the UL30 and UL42 genes, coding respectively for the catalytic and regulatory subunits of the HSV-1 DNA polymerase complex, were cloned as a fusion open reading frame linked through the FMDV2A auto-protease peptide either on a single plasmid (pPol) or inserted through a Gateway reaction in the above mentioned pTF3 plasmid (pTF3Pol) (Figure 3A). Surprisingly, addition of the UL30/UL42 genes to factors encoded by the pTF3 and pRF plasmids strongly increased Rep synthesis as well as the level of AAVtCR replication (Figure 3B and 3C). Similarly, transfection of the pPol plasmid was able to complement, at least partially, the reduced ability of an HSVΔUL30 mutant virus to induce AAVtCR replication as compared with wt HSV-1 infection (Figure 3C). A similar effect was also observed using an HSVΔUL42 mutant virus (Figure S5). Quantification of this effect by qPCR indicated that addition of UL30/UL42 to the other HSV-1 factors (pTF3 and pRF) or to HSVΔUL30 mutant virus induced, on average, a 60- and 11-fold increase in AAV DNA replication, respectively (Figure 3D). To further demonstrate a role for the HSV-1 DNA polymerase, a plasmid encoding for an UL30 protein with a G885A mutation in its catalytic domain was generated. The characterization of this mutant, which was previously documented to lack any detectable DNA polymerase activity [15],[16], indicated that the protein was still able to localize to the nucleus (Figure 4A). In addition, the mutated UL30 protein was not only unable to rescue the growth of the HSVΔUL30 virus (data not shown), but also incapable of stimulating AAVtCR genome replication and Rep protein expression when co-transfected with the pTF3 and pRF plasmids (Figure 4B and 4C). Similar results were also obtained using a different cell clone containing an integrated AAV genome composed of the *rep* gene fused to mCherry and a flag tag (data not shown). Altogether, these results indicated that, in the context of an integrated and latent AAVtCR genome, the optimal combination of HSV-1 factors required to induce efficient Rep synthesis and AAV DNA replication included the ICP0, ICP4, ICP22 immediate-early factors, as well as the UL5/UL8/UL52, UL29 and UL30/UL42 early regulatory proteins. In addition, these results demonstrated for the first time the involvement of the HSV-1 DNA polymerase catalytic activity in *in vivo* AAV replication.

### Visualization of AAV replication centers induced by HSV-1 proteins

**Figure 5:**
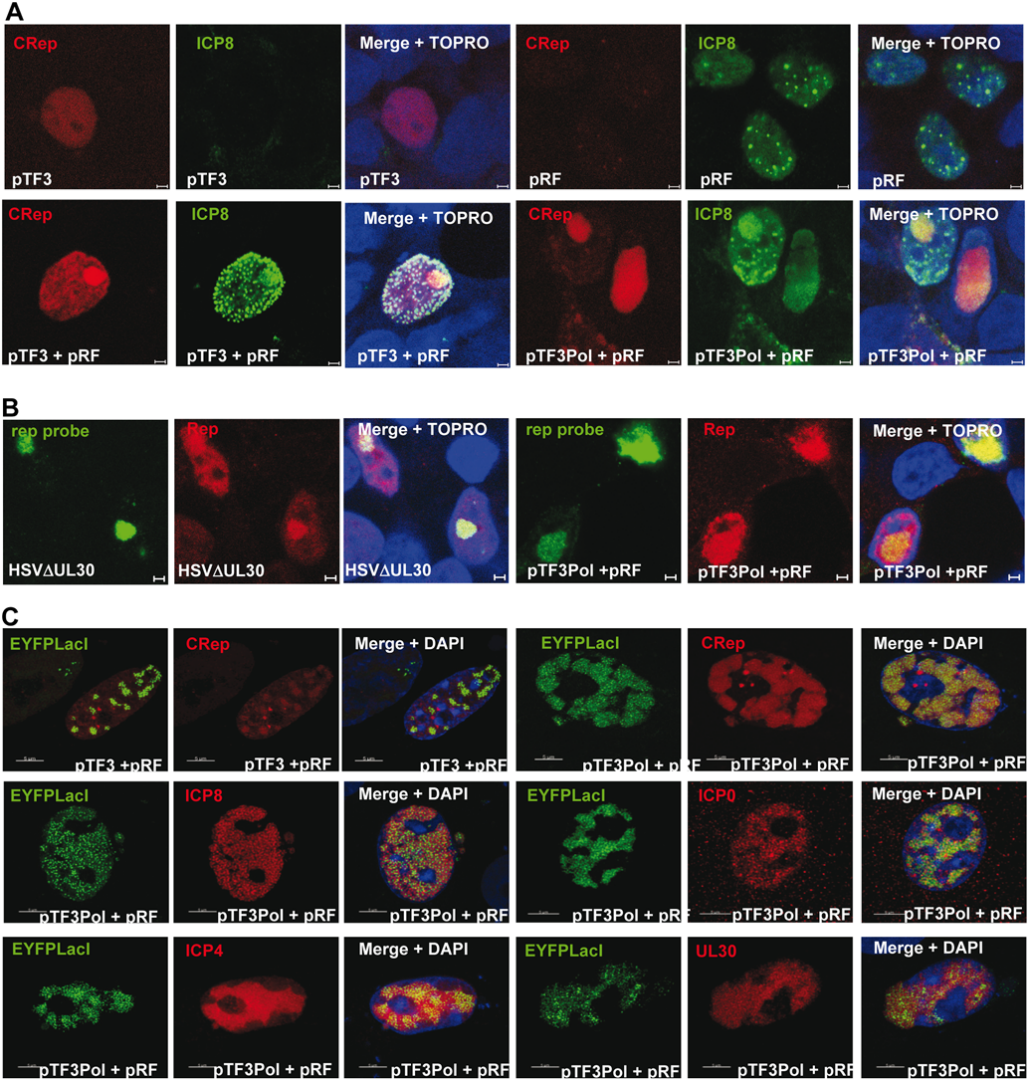
Visualization and analysis of AAVtCR and rAAV replications centers. (A) Co-visualization of Rep and ICP8. HeLaAAVtCR cells, transfected with the indicated plasmids, were fixed and stained with an anti-ICP8 antibody. Cell nuclei were stained with Topro3. The Rep signal was provided by the expression of Rep proteins fused to mCherry (CRep). Bars: 2 µm. (B) Co-visualization by immuno-FISH analysis of AAVtCR DNA and Rep proteins. HeLaAAVtCR cells were either infected with HSVΔUL30 at an MOI of 5 pfu/cell or co-transfected with the pTF3pol and pRF plasmids and fixed 48 or 24 h later, respectively. The AAVtCR DNA was visualized using a combination of four non overlapping DIG-labeled rep probes followed by incubation with an anti-DIG antibody; Rep proteins were detected using an anti-Rep antibody (76.3). Cell nuclei were stained with Topro3. Bars: 2 µm. (C) Co-visualization of rAAV DNA with either Rep, ICP4, UL29, or UL30 proteins. Vero cells were co-transfected with the indicated combinations of HSV-1 plasmid, pAAVlacO, pSV2-EYFP/lacI, and p5CR (upper line) or p5Rep (middle and bottom line). Two days later cells were fixed and either directly observed under a confocal microscope (top line) or stained for detection of ICP8, ICP0, ICP4, and UL30 using antibodies. Cell nuclei were stained with DAPI. CRep: Cherry-Rep. Bars: 5 µm.

Previous studies have demonstrated that in transfected cells, the HSV-1 ICP8 protein and HP complex assemble into discrete foci that can be visualized by a punctuate ICP8 staining [17],[18]. In addition, Rep was demonstrated to interact with ICP8, and to be recruited into ICP8-containing foci formed during wt HSV-1 and AAV co-infection [7],[8]. Consequently, to correlate our results to the formation of AAV replication centers, transfected HeLaAAVtCR cells were examined for the intranuclear localization of the Rep and ICP8 proteins. Consistent with the results presented in Figure 2 and 3, transfection with the pTF3 plasmid led to Rep protein synthesis visualized as a weak diffuse intra-nuclear fluorescence without ICP8 staining whereas transfection of pRF alone led to the typical ICP8 punctuate staining pattern but no Rep synthesis (Figure 5A, upper line). Co-transfection of pTF3 and pRF promoted re-localization of a fraction of the Rep proteins in small and large foci that co-localized with ICP8, clearly evoking pre-replication and replication centers, respectively (Figure 5A, lower left). Expression of the HSV-1 DNA polymerase (UL30/UL42), in addition to the latter HSV-1 factors, resulted in the formation of larger ICP8 foci that co-localized with Rep proteins (Figure 5A, lower right). FISH analysis of these foci with a probe recognizing *rep* DNA showed the presence of AAV DNA co-localizing with Rep proteins both in cells infected with HSV-1 or transfected with pTF3pol/pRF, thus confirming the identification of these foci as true AAV replication centers (Figure 5B). These results confirmed that the co-transfection of two plasmids encoding nine HSV-1 proteins (UL5/UL8/UL52, UL29, UL30/42, ICP0, ICP4 and ICP22) resulted in the formation of AAV replication centers.

Finally, to easily monitor AAV replication in cells, we took advantage of the visualization system previously developed by Fraefel *et al.* [19]. This method relies on the detection of AAV replication by co-transfecting cells with a rAAV vector containing numerous Lac0 binding sites and a second plasmid encoding a fluorescent LacI binding protein. Using this method, rAAV replication foci that co-localized with Rep proteins were observed upon transfection of pTF3 and pRF plasmids. Also, co-expression of the UL30/UL42 genes generated larger replication centers within the nucleus that co-localized with ICP8 (Figure 5C, upper line). Finally, these analyses indicated, for the first time, the co-localization of ICP0, ICP4 and UL30 proteins with replicating rAAV DNA, further supporting their involvement in AAV replication (Figure 5C, middle and lower line).

## Discussion

Wt AAV-2 is defined as a defective virus because it requires the presence of a helper virus to efficiently replicate. Understanding the nature of the helper functions provided by different viruses is essential to identify the cellular factors that control the AAV life cycle. It is also important to provide new tools and strategies for the development of rAAV vectors. The objective of this study was to integrate some previous observations regarding the HSV-1 helper activities and to further explore the requirement for other HSV-1 proteins during early steps in AAV replication. Using a cellular model that mimics AAV latency and composite constructs encoding several HSV-1 proteins, we demonstrated that nine HSV-1 immediate-early and early proteins are required to efficiently sustain the early steps of the AAV replicative cycle including expression of the *rep* gene and viral DNA replication.

### *Rep* gene activation by HSV-1 immediate-early proteins

The present results confirm our previous observation that ICP0 is able to activate *rep* gene expression and also extend this property to the ICP4 protein [13]. In contrast, ICP27 is shown to be dispensable and, in many experiments, inhibitory for Rep protein synthesis. This latter result is in accordance with previous studies indicating that the ICP27-defective HSV-1 strain can be used for high-titer rAAV productions [20],[21]. The main question raised by these observations concerns the mechanism involved in the transactivating effect exerted by ICP0 and ICP4. How *rep* gene repression is maintained during latency is unknown. Current knowledge indicates that ICP0 activates gene expression without binding DNA but rather acts by the modification and/or the degradation of some cellular factors [22]. We have previously demonstrated that the transactivating activity of ICP0 on the p5 promoter required the RING finger domain, responsible for its E3 ubiquitin ligase activity [13],[23]. Therefore, it is possible that the effect of ICP0 on the AAV p5 promoter might be mediated through the specific degradation of some unidentified repressor. In this study, we additionally demonstrated that ICP4 can also activate *rep* gene expression, albeit at a lower level than ICP0, and that it synergistically acts with ICP0 to increase the level of Rep proteins. Such an effect was not unexpected since previous studies had documented that ICP0 and ICP4 can physically interact and cooperatively activate the expression of a variety of HSV-1 and heterologous promoters, including some cellular ones [24]–[27]. The ICP4 protein is essential to activate the expression of HSV-1 early and late genes and it represses its own transcription as well as that of other immediate-early genes [28],[29]. Although repression by ICP4 involves its binding to high affinity sites in the promoters of target genes, the mechanisms of activation remains unclear since strong ICP4 binding sites are not present in many viral promoters [30],[31]. In the case of the *rep* gene, a consensus ICP4 binding site has not been found. More recently, DeLuca and colleagues have shown that the oligomerization of ICP4 molecules was important for the transactivation of promoters containing weak and degenerate binding sites, probably through the recruitment of transcription factors such as TFIID and TAF250 [32],[33]. Therefore, it is possible that the transactivating activity of ICP4 on the p5 promoter is exerted by binding to degenerate and weak binding sites that cannot be readily identified by sequence analysis. In our study, we also documented a slight but reproducible transactivating effect of ICP22. This factor, which is required for the optimal expression of some HSV-1 late genes in some non transformed cell lines, is apparently involved in the alteration of cell cycle regulatory proteins and in the modification of cellular RNA polymerase II [34],[35]. Altogether, these observations suggest that these three HSV-1 factors may act in independent ways: the ICP0 protein appears to be the major transactivating factor that likely exert its activity through the degradation and/or modification of a cellular repressor preventing *rep* gene expression during latency; ICP4 may enhance this effect by binding to some degenerate sites and recruiting cellular transcription factors, and finally, ICP22 may influence *rep* gene expression by acting on the cell cycle proteins.

### Requirement for the HSV-1 immediate-early factors for HP– and ICP8–induced AAV DNA replication

Previous studies indicated that the HSV-1 HP complex and ICP8 were sufficient to induce AAV replication in the absence of any other HSV-1 gene [6],[8],[9]. Our results do not contradict these findings but additionally demonstrate that, in the context of a latent AAV genome, activation of the *rep* gene by ICP0, ICP4, and ICP22 is an essential prerequisite to detect AAV DNA replication upon transfection with plasmids coding for HP and ICP8. These apparently discordant results can be explained by the fact that most of the previous studies were conducted on cells transfected with a plasmid containing the wt AAV-2 genome. In this situation a transcriptional activator was not required since the *rep* gene is transcriptionally active resulting in the synthesis of Rep proteins at a level sufficient to initiate viral DNA replication. In contrast, in our cell model like in other wt AAV-2 latently infected cell lines, the integrated AAV genome is present in a repressed form that can be rescued and replicated upon infection with a helper virus, such as HSV-1. In this context, induction of *rep* gene expression via the activation of the p5 promoter, constitutes the first event before rescue of AAV DNA and DNA replication. As such, we believe that this model is more appropriate to identify all the HSV-1 factors required during all the events of the AAV life cycle, as exemplified in this study by the observed strict dependency upon *rep* gene activation for AAV DNA replication. Importantly, this strict dependency was only partially relieved when cells were transfected with a plasmid coding for the *rep* gene, further indicating that, even in this context, the presence of HSV-1 transcription factors is important to stimulate AAV replication (data not shown).

### Role of the HSV-1 DNA polymerase

Since the initial report describing the HSV-1 helper effect on AAV replication, the role of the HSV-1 polymerase has constituted a matter of debate. Indeed, studies conducted using either HSV-1 strains encoding a thermosensitive DNA polymerase or individual plasmids expressing the HSV-1 early replication factors indicated that the HSV-1 polymerase was not essential to achieve high levels of AAV DNA replication, further suggesting that, in this situation, as in cells co-infected with Ad, AAV exclusively used cellular polymerases to achieve replication of its genome [6]. However, *in vitro* replication assays performed later by Ward and colleagues indicated that the HSV-1 UL30/UL42 complex could replicate AAV DNA [11]. Our results confirm that the HSV-1 UL30/UL42 DNA polymerase complex although not strictly essential, is critical to significantly enhance AAV DNA replication *in vivo*, in particular in the context of transfected cells. Indeed, in cells infected with the HSVΔUL30 mutant, the level of AAVDNA replication was higher than in cells transfected with the minimal set of transcription and replication factors (pTF3+pRF). In addition, during infection the presence of the polymerase significantly increased AAV DNA replication but did not result in a higher level of Rep proteins (see Figure 3D versus 3C). A likely explanation for this latter result is that, in infected cells, in the presence of the HSV-1 DNA polymerase, AAV competes with replicating HSV genomes in particular for the expression of its viral genes. Consequently, enhancing the level of AAV DNA templates does not result in a higher level of Rep proteins synthesis thus explaining why this viral gene was not identified as a critical factor in earlier studies. Altogether, these results highlight a different requirement between infected and transfected cells: on the one hand they confirm that the viral polymerase is not essential, in particular in infected cells; on the other hand, they indicate that in transfected cells the viral polymerase complex, even if not strictly essential, is critical to achieve a high level of replication and Rep proteins synthesis. Finally, in both infected and transfected cells the HSV-1 polymerase catalytic activity is shown to be required to activate AAV DNA replication. This result indicates that the role of the polymerase cannot be restricted to that of a scaffold protein required for the recruitment of cellular factors in AAV replication centers [36],[37].

### A reconstituted model of HSV-1–induced AAV early replication steps

In the case of Ad, five genes have been demonstrated to be sufficient to induce a complete AAV replication cycle leading to the production of infectious particles [3]. In contrast, this and other studies indicated that the HSV-1 helper activities required for AAV gene expression and genome replication, are shared between at least nine genes, thus, reflecting a more complex situation than in the case of Ad.

**Figure 6:**
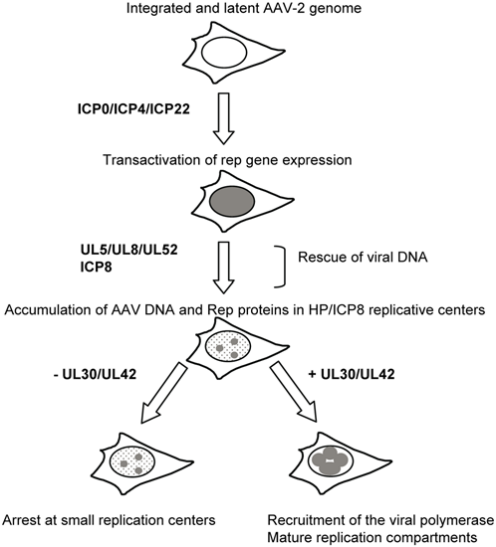
Proposed model for HSV-I induced AAV replication from a latently infected cell line. See the Discussion section for details.

The model describing the sequential events involved in HSV-1- induced AAV replication that arises from these findings is depicted in Figure 6. In cells latently infected with AAV, all the viral genes are in a repressed state. The first event required to activate AAV replication occurs as the transactivation of *rep* gene expression. This step is performed essentially by the HSV-1 immediate-early factors that can act at the transcriptional and/or post-transcriptional level. Induction of Rep synthesis results in a diffuse Rep localization in the nucleus. Rescue of the integrated AAV genome can occur either at this step or after the onset of AAV replication. Indeed, previous studies of this phenomenon have indicated that it can be performed either through the action of cellular nucleases that excise the genome from the cellular chromosome or via a replication-coupled mechanism [38]–[40]. The minimal complex required for AAV DNA replication is composed of the HSV-1 HP and ICP8 proteins that also constitute a scaffold for the formation of replication centers that co-localize with Rep proteins and AAV DNA. In these structures, AAV DNA is replicated through the action of Rep proteins, the HSV-1 helicase (UL5), ICP8, and cellular factors including the DNA polymerases. However, in transfected cells, progression toward the formation of large replication compartments depends upon the presence of the HSV-1 DNA polymerase complex (UL30/UL42) that enhances the level of AAV DNA synthesis. Further analyses are required to determine whether this set of HSV-1 genes is also sufficient to promote the formation of infectious AAV particles that implicates the optimal expression of the *cap* gene and the packaging of AAV genomes.

The main questions raised by this model concern the mechanism and consequence of HSV-1 polymerase-induced AAV DNA replication. Indeed, it remains to be established whether the UL30/UL42-induced replication is conducted in parallel with that induced by cellular polymerases or whether it represents an alternative and exclusive pathway. In addition, it is tempting to speculate that the recruitment of the HSV-1 DNA polymerase in AAV replication centers is involved in the inhibition of HSV-1 replication. Indeed, previous studies have shown that AAV inhibits HSV-1 at the level of DNA replication in a dose dependent manner [41]. Therefore the ability of AAV to use the viral polymerase might explain, at least in part, its capacity to interfere with HSV-1 propagation and promote its own replication. Analysis of the contribution of cellular and HSV-1 polymerase during the establishment of AAV replication centers induced in the presence of HSV-1 particles or genes will help to clarify these issues.

Altogether, these and future studies will be critical to get a better insight into the AAV life cycle and, consequently, introduce new developments in the field of rAAV vectors. Notably, the identification of the HSV-1 helper activities is expected to result in the generation of new tools for the production of rAAV vectors, such as HSV-derived helper plasmids or stable cell lines that could combine elements from different helper viruses to optimally induce the formation of rAAV particles.

## Materials and Methods

### Cells and viruses

African green monkey kidney fibroblasts (Vero), Human cervical epithelial cells (HeLa), and derived cell lines were maintained in Dulbecco's modified Eagle's medium (DMEM, Sigma) supplemented with 10% fetal bovine serum (FCS, HyClone) and 1% penicillin–streptomycin (5,000 U/ml, Invitrogen). The previously described HA-16 cell clone is derived from HeLa cells latently infected wt AAV-2 [42]. The HeLaAAVtCR cell clone was obtained by cotransfecting the pAAVtCR and pPGK-Neo plasmids, the latter conferring resistance to G418 to HeLa cells. Individual G418 resistant clones were screened for the presence of AAV sequences by PCR and then tested for the production of functional and fluorescent Rep proteins upon infection with wt HSV-1. The HSV-1 viruses used in the study were wt HSV-1 (17 syn+), HSVΔUL30 (HP66) provided by D. Coen (Harvard University, Boston, MA, USA). Wt and mutant HSV stocks were produced and titrated by standard procedures on Vero or PolB3 cells (provided by C. Hwang, SUNY Health Science Center, Syracuse, NY, USA), respectively.

### Plasmids

The pAAVtCR plasmid (Figure 2) was obtained by inserting the mCherry ORF from pRSETmCherry (provided by Y. Tsien) and the calmodulin and streptavidin binding peptide (CBP and SBP) tags from pNTAP-A (Interplay Mammalian TAP system, Stratagene) in a modified AAV-2 genome derived from the pSSV9 plasmid [43] from which the *cap* gene had been removed and replaced with the Bovine Growth Hormone (BGH) polyadenylation site (polyA). For the visualization of rAAV vector replication, the *rep* gene was either expressed from p5CR, encoding mCherry [44] fused to the N terminus of AAV Rep68/78 under the control of the p5 promoter and unmodified Rep52/40 under the control of the p19 promoter, or from p5Rep producing unmodified Rep proteins under the control of the AAV p5 promoter.

The HSV-1 plasmids used for this study are described in Figure 3. They were obtained using the Multisite Gateway® Pro System (Invitrogen) that uses a cloning method based on the bacteriophage lambda site-specific recombination system to facilitate the efficient construction of plasmids containing two to four DNA elements [45]. The resulting HSV-1 plasmids contain three or four HSV-1 cDNAs flanked by heterologous promoters and polyadenylation signals (polyAs) and by specific Gateway recombination signals. For the construction of these plasmids, first, four combinations of promoters and polyA were cloned between Xho I and Sac I sites of psp72 (Promega) to give rise to preA, preB, preC and preD plasmids. These plasmids contained the Cytomegalovirus (CMV) promoter and SV40 polyA, the HSV-1 thymidine kinase (TK) promoter and polyA signal (TKpA), the murine phosphoglycerate kinase-1 (PGK) promoter and the rabbit β-globin polyA (βGpA), the short EIF1-α promoter (sEIFα) and a synthetic polyA (spA), respectively. The primers used for this step (sequence available upon request) created a PacI-AvrII-XbaI multiple cloning site between the promoter and polyA signal and inserted two Gateway recombination sites flanking the expression cassette (Invitrogen). The preA, preB, preC and preD contained the B1/B5r, B5/B4, B4r/B3r and B3/B2 Gateway recombination signals and CMV/SV40, TK/TKpA, PGK/βGpA, and sEIFα/spA promoter/polyA, respectively. To generate entry plasmids suitable for use as substrates in a Gateway LR reaction, the preA, preB, preC, and preD vectors were recombined using BP Clonase II with the Multisite Gateway Pro Donor vectors pDONR221 P1-P5r, pDONR221 P5-P4, pDONR221 P4r-P3r, and pDONR221 P3-P2, respectively. The resulting entry plasmids were named pA, pB, pC, and pD and contained a PacI-AvrII-XbaI cloning site where the HSV-1 genes were then inserted. The ICP0 and ICP4 coding sequences were obtained from plasmids pCi110 (provided by R. Everett) and pCMVICP4, respectively. The other HSV-1 genes were amplified by PCR from the HSV-1 containing cosmids cos6, cos14, cos 28, cos48 and cos56 [46] using the Phusion High-Fidelity DNA polymerase (Finnzymes). The positions of the amplified sequences on the wt HSV-1 genome (GenBank no. NC_001806) were: UL5, 15,172–12,471 bp; UL8, 18,217–20,507 bp; UL52, 109,029–112,231 bp; UL29: 62,091–58,431 bp; UL54 (ICP27), 113,689–115,277 bp; US1 (ICP22), 132,608–133,919 bp. The UL30 and UL42 coding sequences (UL30, 62,755–66,512, and UL42, 93,113–94,617 bp) were fused using the Foot and Mouth Disease Virus autoprotease sequence 2A (FMDV2A in bold) using the following primers: UL30rev-: 5′-tttttt**aagcttaagaaggtcaaaattcaacagctg**tgctagagtatcaaaggctcta-3′; UL42for-: 5′-ttttt**aagcttgcgggagacgtcgagtccaaccccgggccc**atgacggattcccctggcgg-3′. The Hind III site used to join the sequences is underlined. The resulting coding sequence was cloned in pB and pC giving rise to pBpol and pPol plasmids. Each individual plasmid was sequenced to check for the absence of mutations and a subset of these individual plasmids (pD-ICP4, pB-CP27, pB-UL29, pB-pol, pPol, and p-UL52) were tested for their ability to complement the growth of HSV-1 strains with a mutation in the corresponding genes (Figure S4). Finally, combinations of four entry plasmids were assembled into a unique construct by a single Gateway® LR reaction using pDEST-14 (Invitrogen) as a destination vector. Reactions performed with pA-UL5, pB-UL29, pC-UL8 and pD-UL52 gave rise to pRF; with pA-ICP0, pB-pol, pC-ICP22, and pD-ICP4 to pTF3pol. pTF3 was obtained from pTF3pol after a BP reaction with pDONR221 P5-P4 which removed the *pol* gene. The properties of these three plasmids were demonstrated to be similar to those of the corresponding individual plasmids (data not shown). The previously described G885A mutation into region I of the HSV-1 DNA polymerase UL30 was introduced into the plasmid pPol by site-directed mutagenesis using Quikchange XL Mutagenesis kit (Stratagene) [15],[16]. Primers used were 5′-cgcatcatctacgcggacacggactcc-3′ and its reverse complement (mutation underlined). The presence of the mutation in the pPol plasmid was checked by sequencing and the resulting plasmid was named pPolG885A.

### Southern blot

For this and other analyses cells were seeded in a 6-well plate at a density of 5×10^5^ cells per well. Two days later, the cells were transfected using standard calcium-phosphate procedure and either fixed or harvested 48 h later for genomic DNA extraction. For the analysis, 2 to 4 µg of total DNA were digested with EcoR V or Pst I, run on 1% agarose gel and transferred to a Hybond N+ membrane (GE Healthcare). The membrane was then hybridized overnight to a digoxigenin (DIG)-labeled probe in EasyHyb solution (Roche). DIG- labeled probes were synthesized using the DIG PCR Probe Synthesis kit (Roche) following the manufacturer's instructions. Probe size and DIG incorporation were checked on 1% agarose gel. The 513 bp gfp probe used for Southern Blot was obtained by PCR with primers GFP-F (5′-cctgaagttcatctgcacca-3′) and GFP-R (5′-ttctcgttggggtctttgct-3′). The 902 bp rep probe was synthesized using primers Rep2 (5′-cgagattgtgattaaggtcc-3′) and Rep4 (5′-ccgcatattggggatcgtac-3′). After overnight hybridization the membrane was processed following the manufacturer's instructions, incubated 5 min at RT with CDP-star (Roche), and then exposed to an autoradiography film.

### Quantitative PCR

Primers used for the qPCR reaction were: REP-F (5′-gcaagaccggatgttcaaat-3′) and REP-R (5′-cctcaaccacgtgatccttt-3′); ChrX-F (5′-gacagtcagccgcatcttctt-3′) and ChrX-R (5′-agttaaaagcagccctggtga-3′). For all reactions, 10 µL of 2× Platinum SYBR Green qPCR Supermix Uracil DNA Glycosydase (UDG) (Invitrogen) were used in a final volume of 20 µL (in a 96 well tray, Applera), and a final concentration of 400 nM of each primer pair. Approximately 15 ng of DNA were added in 5 µL of solution. Reactions were always set up in duplicate. Each PCR was performed as follows: initial uracil DNA glycosylase decontamination at 50°C for 2 min followed by a 10 min hot-start denaturation at 95°C, and 40 amplification cycles (15 s at 95°C, 30 s at 60°C). The melting temperature of the final double-stranded DNA products was determined by gradual heating from 60°C to 95°C over 20 min. All qPCR reactions were performed with the ABI Prism 7000 Sequence Detection System apparatus (Applera). Absolute amounts of Rep and ChrX amplicons in arbitrary units were determined using dilutions of genomic DNA from HeLaAAVtCR cells mock transfected with a GFP expressing plasmid as a standard. Calculations were done as described by Pfaffl *et al.* [47] The data are expressed as Rep/Chr.X ratio, that was fixed at 1 for the HeLaAAVtCR mock transfected cells.

### Western blot analysis

The cells were collected, washed in phosphate-buffered saline (PBS) and lysed with RIPA buffer (20 mM Tris-HCl pH 7.4, 50 mM NaCl, 1 mM EDTA, 0.5% Nonidet P-40, 0.5% deoxycholate, and 0.5% SDS) in the presence of a cocktail of protease inhibitors (Roche). Proteins were loaded on 10% SDS poly-acrylamide gels and then transferred to nitrocellulose membranes (GE Healthcare). After saturation, the membranes were incubated overnight at 4°C with the appropriate antibody diluted in blocking buffer. The anti-ICP4 (ab6514), and anti-ICP0 (ab6513) mouse monoclonal antibodies were purchased from Abcam (Cambridge, United Kingdom) and used at a 1/2500 and 1/1000 dilution, respectively. The anti-Rep 303.9 antibody mouse monoclonal antibody was used at a 1/20 dilution. The anti-tubulin (T5168, SIGMA) mouse monoclonal antibody was used at a 1/2000 dilution. After PBS washes, a horseradish peroxidase-conjugated anti-mouse antibody (Dako) was applied to the membranes at a 1/20000 dilution for 1 h at RT. Finally, the membranes were incubated for 5 minutes with the enhanced chemiluminescence reagent (West Dura, Pierce) and exposed to an autoradiography film. When necessary, the membranes were stripped for 30 min at 60°C in Tris, 62.5 mM, 2% SDS, 0.1 M β-mercaptoethanol, pH 6.8 and then probed with another primary antibody.

### Immunofluorescence analysis

The cells were seeded on 10 mm-round glass slides, and all steps were performed at RT unless otherwise indicated. The cells were fixed in PBS, 4% paraformaldehyde (PFA) for 10 min and then permeabilized with PBS, 0,5% Triton X-100 for 30 min. The cells were incubated with PBST (PBS, 4% bovine serum albumin, 0,2% Tween) for 30 min before incubation for 1 h at RT with the appropriate antibody diluted in PBS-T. The monoclonal anti-ICP8 mouse antibody (ab20194, Abcam) was diluted 1/200 and the monoclonal 76.3 anti-Rep mouse antibody was used pure. Next, the slides were washed in PBST and then incubated with an Alexa Fluor 488 donkey anti-mouse antibody (Invitrogen) diluted 1/2000 in PBS-T for 1 h in the dark. After two PBST and two PBS washes, the slides were incubated for 5 min with TOPRO-3 used at a 1/5,000 dilution (Invitrogen). After extensive washes, the coverslips were mounted onto glass slides with Fluoromount-G (CliniSciences). Images were collected on a Confocal Axioplan2 LSM510 (Zeiss).

### Immuno-FISH analysis

Cells were fixed and permeabilized as indicated above. After incubation with 0,1 N HCl in PBS for 3 min, the cells were treated 1 h at 37°C with 100 µg/mL of RNAse A in 2×SSC followed by two 2×SSC washes. The slides were then equilibrated in 2×SSC, 50% formamide (pH 7) for 30 min, and then eventually stored at 4°C. FISH was performed using a mixture of four non overlapping DIG-labeled probes of 400–500 bp covering the *rep* gene (sequences available upon request). DIG labeled probes were precipitated with sodium acetate and ethanol, resuspended in FISH Hybridization buffer (2×SSC, 50% Formamide, 10% Dextran Sulfate, pH 7) and then added to the coverslips that were sealed on glass slides. After denaturation 3 min at 75°C, the slides were incubated overnight at 37°C. After post-hybridization washes, the samples were saturated with PBST for 30 min. The primary sheep anti-DIG antibody (Roche) was diluted 1/4000 and the anti-Rep antibody (76.3) was used undiluted. The secondary donkey anti-sheep Alexa 488 and donkey anti-mouse Alexa 555 antibodies (Invitrogen) were used at a 1/4000 and 1/2000 dilution, respectively in PBS-T. After incubation with the primary and secondary antibodies for 1 h at 37°C, the samples were washed and the nuclei counterstained with Topro-3 at a 1/5000 dilution in PBS (Invitrogen). Preparations were mounted in Fluoromount-G (CliniSciences). Images were collected using a Confocal Axioplan2 LSM510 (Zeiss).

### Visualization of rAAV replication centers

The visualization of rAAV replication was performed as previously described [19]. Briefly, the day before transfection, Vero cells were seeded on round 12 mm cover glasses in 24-well plates at 10^5^ cells/well. Cells were transfected using Lipofectamine Plus reagent (Invitrogen). The amounts of individual plasmids used for transfection were as follows: pAAVlacO, 25 ng; pSV2-EYFP/lacI, p5Rep, and p5CR, 10 ng. Helper functions for rAAV replication were provided by co-transfection with 50 ng of pTF3pol and pRF, respectively. Cells were fixed 48 hours after transfection and processed for immunofluorescence. The antibodies used for these analyses were: anti-ICP0 (Abcam 6513), anti-ICP4 (Advanced Biotechnologies 102-124-1), anti-ICP8 (Abcam 20193), and anti-UL30 (mABC-4, a kind gift from C. Knopf). Nuclei were stained with DAPI. Confocal microscopy was performed essentially as described previously [19].

### Accession numbers

Rep68: NC_001401; YP_680422; (GeneID: 4192013)

Rep78: NC_001401; YP_680423; (GeneID: 1489608)

Rep40: NC_001401; YP_680424; (GeneID: 4192014)

Rep52: NC_001401; YP_680425; (GeneID:1489607)

UL5: NC_001806; NP_044606; (GeneID:2703420)

UL8: NC_001806; NP_044609; (GeneID:2703432)

UL52: NC_001806; NP_044655; (GeneID:2703423)

ICP8 (UL29): NC_001806; NP_044631; (GeneID:2703458)

ICP22 (US1): NC_001806; NP_044663; (GeneID:2703435)

ICP27 (UL54): NC_001806; NP_044657; (GeneID:2703426)

ICP0 (RL2): NC_001806; NP_044660; (GeneID:2703390)

ICP4 (RS1): NC_001806; NP_044662; (GeneID:2703392)

UL30: NC_001806; NP_044632; (GeneID:2703462)

UL42: NC_001806; NP_044644; (GeneID:2703407)

Human Chr.X: NT_079573

## Supporting Information

Figure S1 Analysis of AAVtCR genome copy number and site-specific integration in HeLaAAVtCR cells.
(0.96 MB TIF)

Figure S2 Hela cells were transfected with the indicated plasmids and, 6 hours later, infected with wt AAV-2 particles at an MOI of 1000 particles/cell.
(0.66 MB PPT)

Figure S3 HA-16 were either left untreated (-) or infected at the indicated MOIs with either wt HSV or HSVΔICP0 (dl1403).
(0.37 MB TIF)

Figure S4 Functional activity of the pPol plasmid.
(1.82 MB TIF)

Figure S5 Both UL30 and UL42 HSV polymerase subunits enhance AAVtCR genome replication.
(1.31 MB PDF)

Figure S6 Quantification of the effects of HSV-1 immediate early factor on *rep* gene expression.
(0.31 MB TIF)
