# Supplementary figures and images for: Definition of Herpes Simplex Virus Type 1 Helper Activities for Adeno-Associated Virus Early Replication Events
Source: PLoS Pathog. 2009 Mar 13;5(3):e1000340. doi: 10.1371/journal.ppat.1000340 (PMC2650098; doi:10.1371/journal.ppat.1000340)

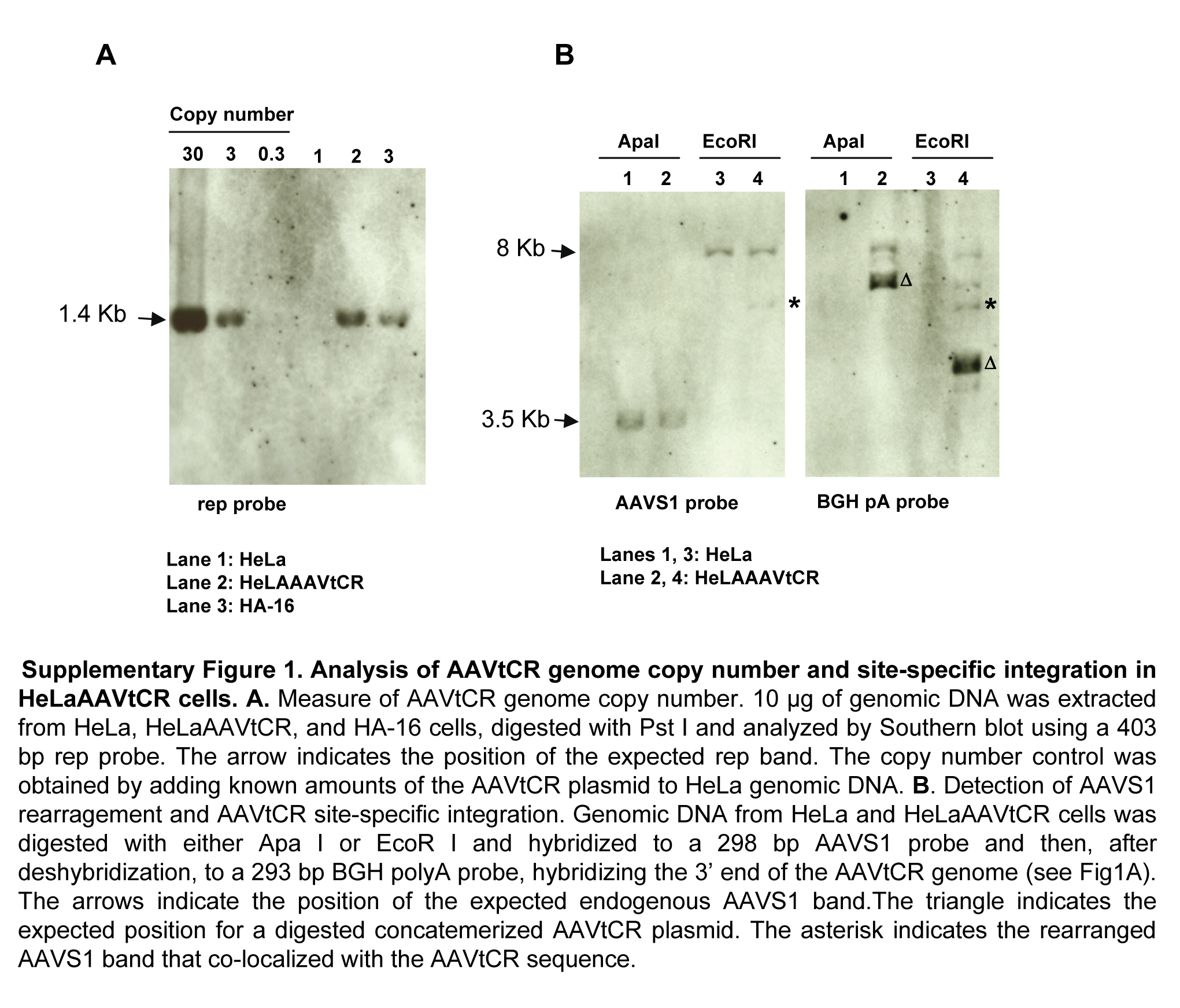

Supplement: Figure S1 — Analysis of AAVtCR genome copy number and site-specific integration in HeLaAAVtCR cells. (0.96 MB TIF) [file ppat.1000340.s001.tif]

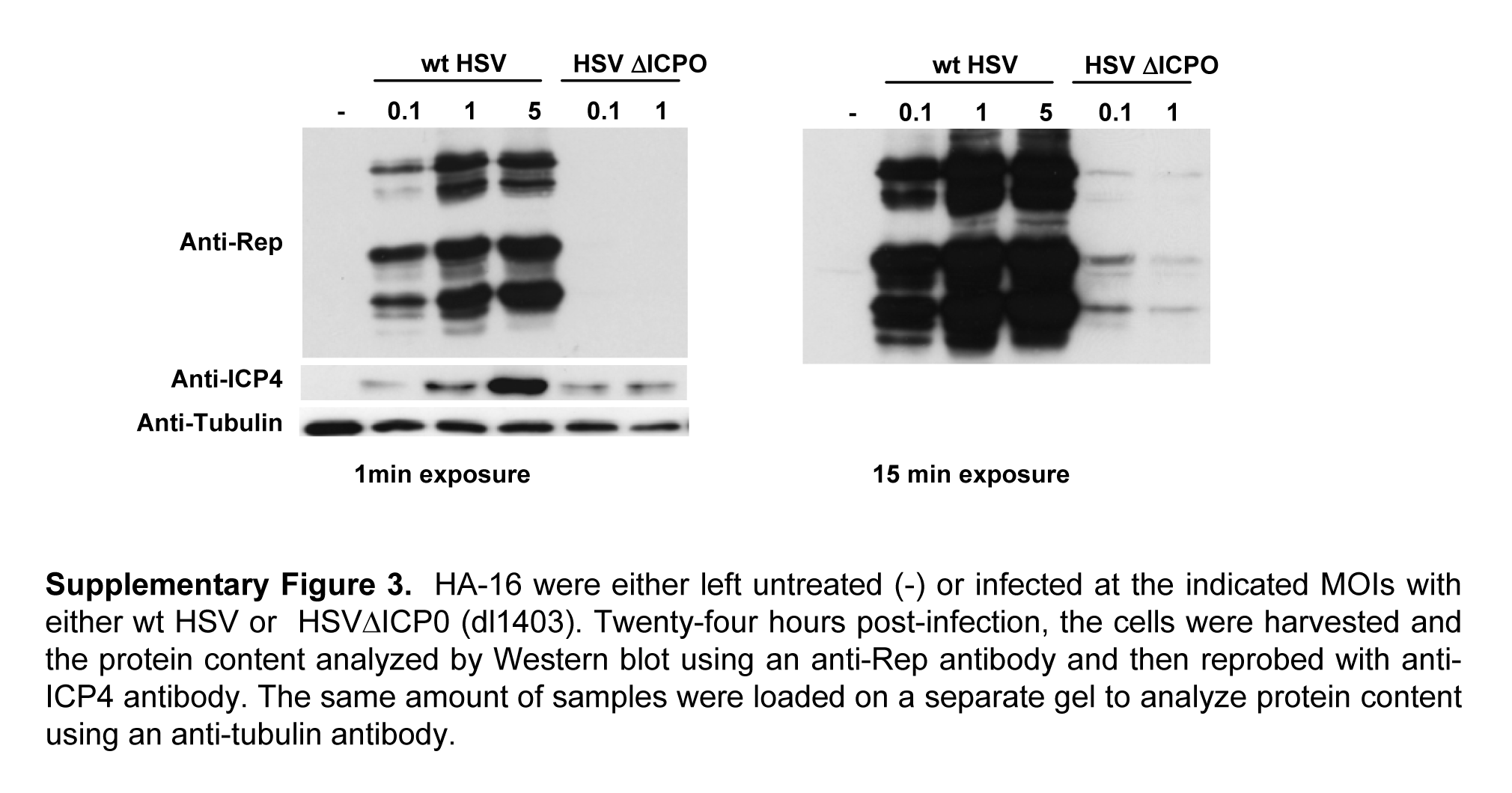

Supplement: Figure S3 — HA-16 were either left untreated (-) or infected at the indicated MOIs with either wt HSV or HSVΔICP0 (dl1403). (0.37 MB TIF) [file ppat.1000340.s003.tif]

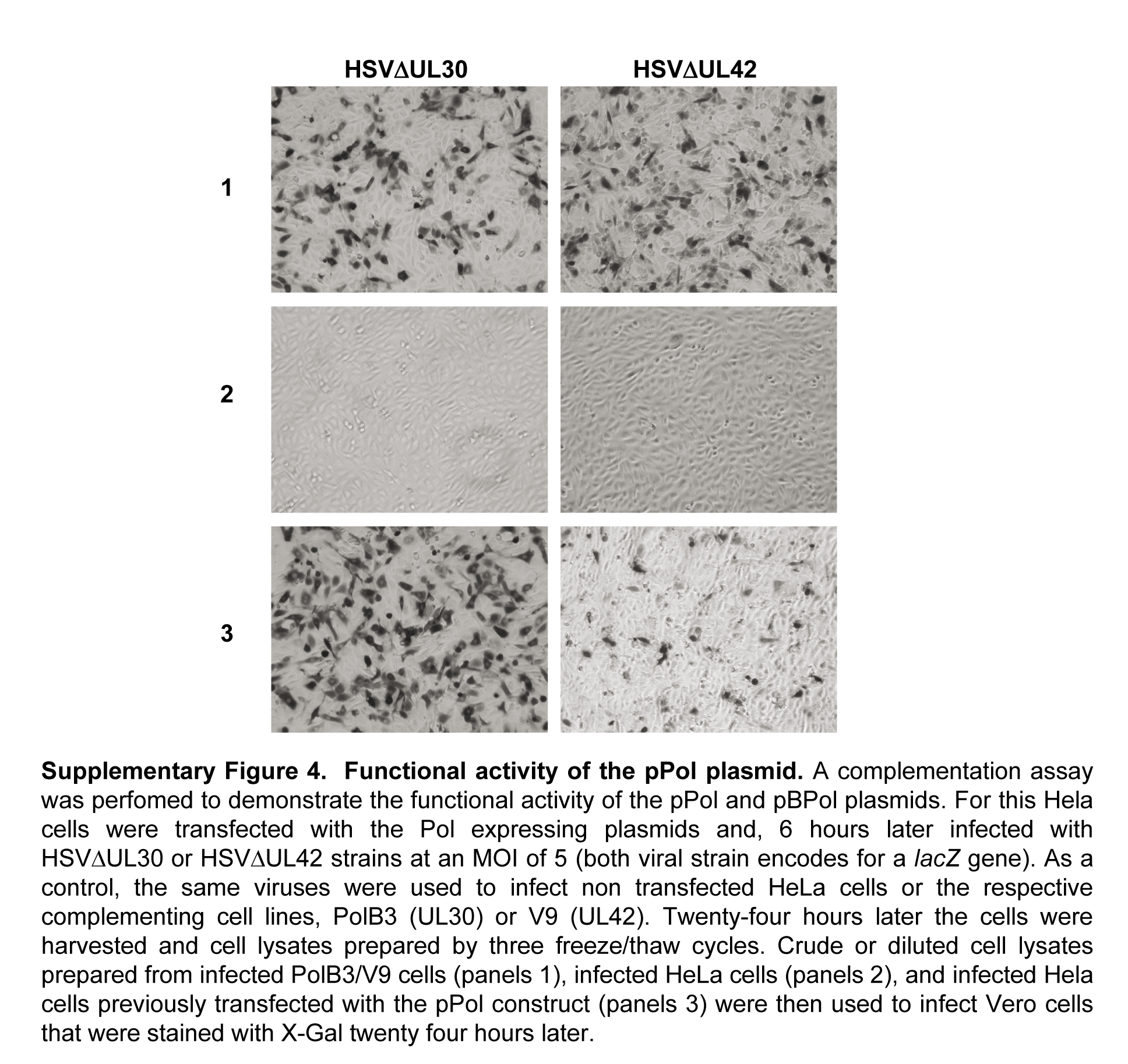

Supplement: Figure S4 — Functional activity of the pPol plasmid. (1.82 MB TIF) [file ppat.1000340.s004.tif]

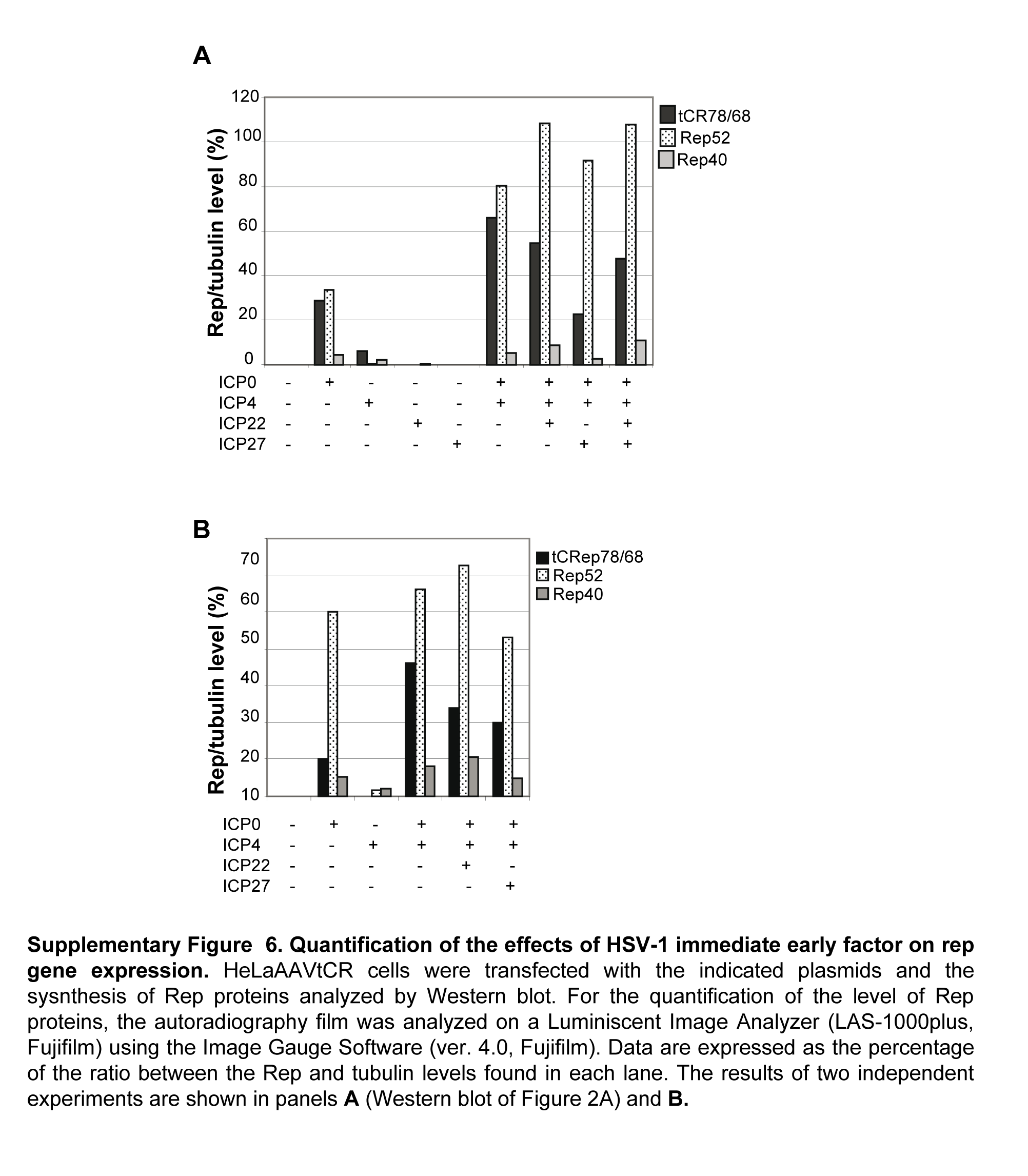

Supplement: Figure S6 — Quantification of the effects of HSV-1 immediate early factor on rep gene expression. (0.31 MB TIF) [file ppat.1000340.s006.tif]
