# Supplementary material for: Definition of Herpes Simplex Virus Type 1 Helper Activities for Adeno-Associated Virus Early Replication Events
Source: PLoS Pathog. 2009 Mar 13;5(3):e1000340. doi: 10.1371/journal.ppat.1000340 (PMC2650098; doi:10.1371/journal.ppat.1000340)
Supplement: Figure S2 — Hela cells were transfected with the indicated plasmids and, 6 hours later, infected with wt AAV-2 particles at an MOI of 1000 particles/cell. (0.66 MB PPT) [file ppat.1000340.s002.ppt]

## Slide 1
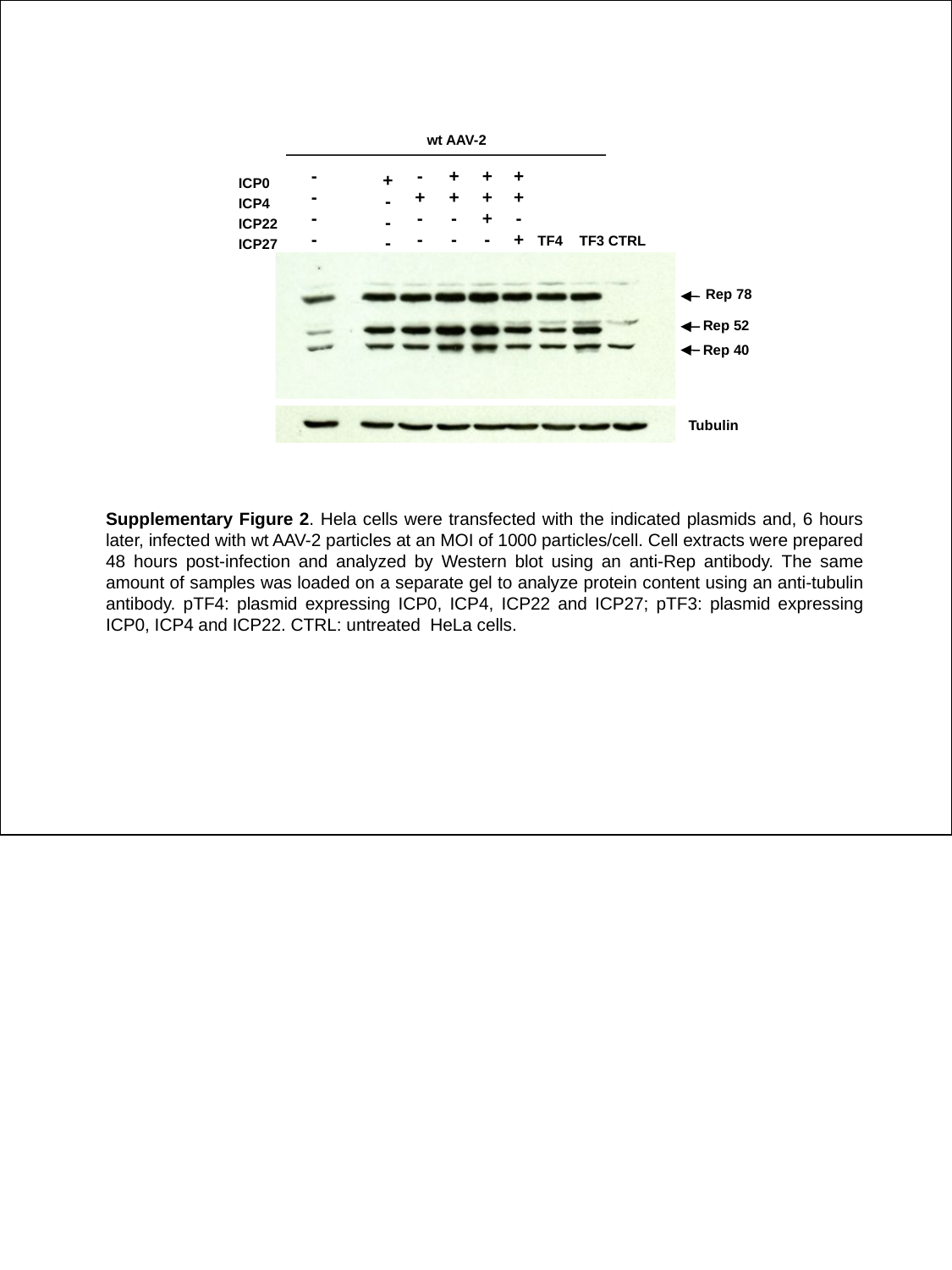

wt AAV-2
-
-
-
-
-
+
-
-
+
+
-
-
+
+
+
-
+
+
-
+
+
-
-
-
ICP0
ICP4
ICP22
ICP27
TF3
CTRL
TF4
Rep 78
Rep 52
Rep 40
Tubulin
Supplementary Figure 2. Hela cells were transfected with the indicated plasmids and, 6 hours later, infected with wt AAV-2 particles at an MOI of 1000 particles/cell. Cell extracts were prepared 48 hours post-infection and analyzed by Western blot using an anti-Rep antibody. The same amount of samples was loaded on a separate gel to analyze protein content using an anti-tubulin antibody. pTF4: plasmid expressing ICP0, ICP4, ICP22 and ICP27; pTF3: plasmid expressing ICP0, ICP4 and ICP22. CTRL: untreated HeLa cells.
