# Supplementary material for: Definition of Herpes Simplex Virus Type 1 Helper Activities for Adeno-Associated Virus Early Replication Events
Source: PLoS Pathog. 2009 Mar 13;5(3):e1000340. doi: 10.1371/journal.ppat.1000340 (PMC2650098; doi:10.1371/journal.ppat.1000340)
Supplement: Figure S5 — Both UL30 and UL42 HSV polymerase subunits enhance AAVtCR genome replication. (1.31 MB PDF) [file ppat.1000340.s005.pdf]

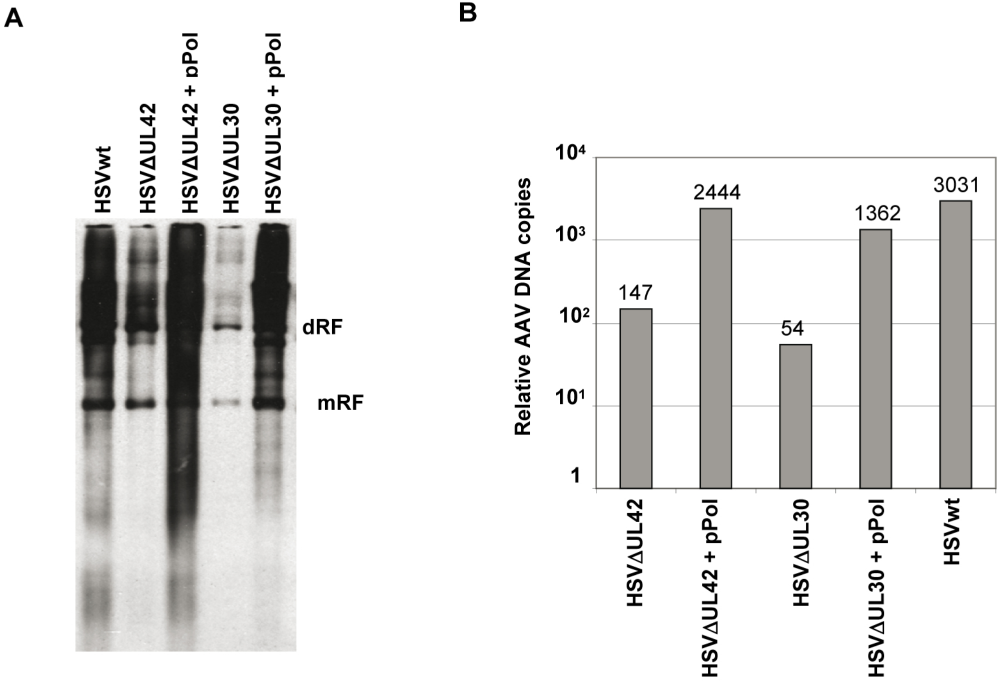

Supplementary Figure 5. Both UL30 and UL42 HSV polymerase subunits enhance AAVtCR genome replication. HeLaAAVtCR cells were infected with wt HSV-1, HSVΔUL42 or HSVΔUL30 (MOI of 5 pfu/cell) after transfection with pPol plasmid where indicated. **A.** Genomic DNA was extracted and analyzed by Southern blot using a rep probe. **B.** Analysis of AAVtCR genome replication by qPCR using primers located in the AAV *rep* gene.
